# Svapnia

**Published:** 1870-01

**Authors:** John M. Bigelow

**Affiliations:** Detroit, Mich.


### SVAPNIA.

PROF. N. S. DAVIS, M.D.,

*Dear Sir:*—Will you excuse me with a few words of explation with regard to your Report on Drugs and Medicines, made to the Illinois State Medical Society in May last, which only a day or two since came under my observation?

In reference to the disadvantage of svapnia, you say: "*We see no reason why* each physician should not combine morphia, codeia, and narceia, in just such proportions as his patients may need, if there is any advantage to be derived from their combination."

Now, permit me, respectfully, to point out two pretty strong reasons why they cannot, or do not.

1st. In the first place, not one of the constituents of opium, excepting morphia and narcotine, are to be found in the market, and narcotine is well known to possess no anodyne or narcotic property whatever. If the physician cannot get the article how is he to make the combination, no matter how much his desire, or the urgency of his patient's case may be?

2d. I believe Messrs. Morson & Son, of London, and T. &

H. Smith, of Edinburgh, have separated narceia, codeia, niconine, and cryptopia, in purity, by which those who are fond of experiments may obtain them for those purposes, but they are precluded from common use in practice, by their extravagant cost. Dr. Hurley informs us that at present four or five tons have yielded only the same number of ounces of cryptopia. Narceia, I believe, has been sold here, when specially ordered, at 50 to 75 cents per grain. The other principals also in nearly the same proportion.

Dr. Hurley, in his excellent work on the "Old Vegetable Neurotics," discusses the question—which, however, was before decided affirmatively, in the every-day practice of physicians—are there many persons so peculiarly sensitive to nervous impressions that the exhibition of opium and morphia, in ordinary dose, unguarded by controlling medicines or influence, will be productive of distress and danger?

The result of his experiments upon the animal system reveals the fact that the same idiosyncrasy is found to exist in both the horse and the dog.

In his analysis of the effects of the different constituents of opium, he clearly defines the properties of each, and then comes to the conclusion that they are all members of the same family—all having characters in common, but differing greatly, only in degree, in having two opposite properties, to wit: 1st, excitant, which, in excess, runs on to convulsions, and tetanus; 2d, depressant, which, sufficiently intensified, merges in hypnosis, and all in excess end in profound narcotism and death.

Morphia and thebaina are the agents more marked than the other constituents, in the production of these opposite effects; morphia at the head of the class of hypnotis, and thebaina at the head of the tetanizing forces. The effects of either even in slight excess upon those peculiarly susceptible to their morbid influences are productive of distress, if not of danger to life. Hence it is well to have the system guarded from the deleterious effects that possibly may accrue; and Dr. Hurley, therefore says we must never give morphia by injection under the skin, without some protection from these effects, in persons with whom we have reason to suspect such ill consequences may result? He recommends the use of atropia. Why? Not to fulfil any special indications in the combat of disease, but to guard against the deleterious effects of the morphia.

One of the peculiar effects of morphia is the derangement of the vagus, or pneumogastric nerve, producing violent retching, vomiting, and nausea. Another effect, probably in a great measure depending secondarily upon its effect upon this nerve, is that the whole respiratory function is depressed—these effects in the highly excitable nervous diathesis, or the peculiar anti-morphine idiosyncrasy, are so magnified as to become dangerous. In crude opium and laudanum in these cases, we have the convulsive and tetanizing properties of thebaina and cryptopia to contend against, in which danger is produced from that source.

Svapnia fulfils more directly the indications sought for by Dr. Hurley, in the combination of atropia, and morphia, with, we think, much less danger and greater convenience. In it, the thebaina and cryptopia are entirely eliminated, and the depressant effect of the morphia upon the pneumogastric nerve, counteracted by its union with the less excitant properties of the natural constituents of opium, namely: niconine, codeia, and narceia. In this respect, svapnia must be considered a simple remedy, or pure opium, so eliminated of its poisonous constituents of thebaina, and cryptopia, as to render its exhibition, when its anodyne and hypnotic effects are needed, less dangerous, even, than when morphia is conjoined with atropia.

JOHN M. BIGELOW.

*Detroit, Mich., December 3d, 1869.*

---

Permanganate of Potassa.—A solution of permanganate of potassa will be found a valuable remedy in the treatment of disease of the antrum, in the proportion of 5 parts to 100 of water. We have recently applied this solution, in the form of an injection, in several cases of disease of this cavity; and in a short time after its use was commenced, the disagreeable odor was diminished and the good effects of the agent apparent. It is also valuable in ozaena and other affections similar in character.—*American Journal of Dental Science.*

